# Circulatory lipid signature in response to short-term testosterone gel treatment of healthy young females

**DOI:** 10.1038/s41598-025-92690-6

**Published:** 2025-03-29

**Authors:** Olivier Salamin, Justin Carrard, Toni Teav, Rebecca Borreggine, Jessica Medina, Raul Nicoli, Tiia Kuuranne, Martial Saugy, Nelly Pitteloud, Hector Gallart-Ayala, Julijana Ivanisevic

**Affiliations:** 1 Swiss Laboratory for Doping Analyses, University Center of Legal Medicine, Lausanne and Geneva, Lausanne University Hospital and University of Lausanne, Lausanne, Switzerland; 2 Division of Sport and Exercise Medicine, Department of Sport, Exercise and Health, University of Basel, Grosse Allee 6, 4052 Basel, Switzerland; 3 SportAdo Centre, Children and Adolescent Surgery, Woman-Mother-Child Department, Lausanne University Hospital, Lausanne, Switzerland; 4 Metabolomics Unit, Faculty of Biology and Medicine, University of Lausanne, Quartier UNIL-CHUV, Rue du Bugnon 19, 1005 Lausanne, Switzerland; 5 Center of Research and Expertise in Anti-Doping Sciences - REDs, Institute of Sport Sciences, University of Lausanne, 1015 Lausanne, Switzerland; 6 Service of Endocrinology, Diabetology, and Metabolism, Lausanne University Hospital, Lausanne, Switzerland

**Keywords:** Testosterone gel, Lipidomics, Circulatory lipid profile, Healthy women, Short-term treatment, Endocrine system and metabolic diseases, Endocrine reproductive disorders

## Abstract

The impact of testosterone administration on the circulating lipidome in females remains unexplored, despite its relevance to understanding metabolic disorders like polycystic ovary syndrome (PCOS). This study addresses this gap by examining the effects of testosterone gel on the plasma lipidome of healthy women over three menstrual cycles. A cohort of 14 women aged 22–37 years with regular cycles was analyzed, with plasma samples collected at baseline, during peak testosterone levels (D45), and post-treatment (D59, D80). Testosterone gel treatment lasted 28 days, administered between day 29 and day 57 of the study. Using a deep-targeted lipidomic approach, 597 lipids were quantified to provide a detailed profile of the lipidome and capture subtle changes in lipid species and their associations with testosterone fluctuations. Extensive profiling revealed a significant decrease in 17 lipid species, especially ether- and ester-linked lysophosphatidylcholines (LPC), at peak testosterone. These lipid reductions were strongly negatively correlated with free and total testosterone, as well as dihydrotestosterone (DHT), and positively correlated with SHBG levels. Notably, intra-individual lipid variability was consistently lower than inter-individual variability, indicating a highly personalized lipidome regulation. Despite testosterone-induced changes, overall plasma lipidome alterations were minimal, suggesting mechanisms that maintain lipid homeostasis. This study highlights the complex interplay between testosterone and lipid metabolism in women. The minimal overall lipidome changes and high inter-individual variability point to the need for further research to assess the clinical relevance of these findings, particularly in hyperandrogenic conditions like PCOS.

*Clinical Trial Registration number*: This study was registered on https://www.isrctn.com/ (ISRCTN10122130) on 09/01/2019.

**Supplementary Information:**

The online version contains supplementary material available at 10.1038/s41598-025-92690-6.

## Introduction

Testosterone is the sex hormone responsible for the development of males’ secondary sexual characteristics as well as the maintenance of muscle mass and bone density. Testosterone also regulates multiple other physiological processes, including the production of red blood cells, carbohydrate and lipid metabolism, and sex drive regulation. Testosterone in males primarily originates from the testes, while in females, only limited amounts are produced by the ovaries. Instead, the adrenal gland and peripheral tissues contribute significantly to the testosterone pool in females by converting androgen precursors^1^. The circulating level of testosterone in women is seven to eight times lower than in men. Notably, the production of testosterone declines with age in both sexes^2^. This slow but progressive age-related decline has been associated with a greater risk of developing various conditions such as osteoporosis, metabolic syndrome or neurodegenerative diseases especially in men, while data are scarce for women^3^.

Testosterone replacement therapy using diverse exogenous testosterone formulations has been widely used in males for the treatment of hypogonadism and other non-endocrine diseases such as anemia, muscular dystrophy, or dermatological diseases^4^. In women, testosterone has also been extensively applied to treat hypoactive sexual desire disorder (HSDD) despite the lack of an approved formulation^5^. Testosterone constitutes a vital element of female sexuality and is associated with greater well-being, improved energy, and vitality. Accordingly, several studies have demonstrated that transdermal testosterone administration in small doses had beneficial effects on sexual function, mood, and well-being, as well as anxiolytic effects in premenopausal and menopausal women^6–9^. The administration of exogenous testosterone has also been used in the context of sport due to its ergogenic effect^10^. Given the lower basal testosterone concentration in women, an increase in the circulating testosterone level is supposed to generate a significant boost in performance. Exogenous testosterone is particularly effective in strength sports, dose-dependently enhancing muscle mass and lean body mass^11^. In addition to its anabolic action, testosterone administration in small and repeated doses can reduce fatigue, stimulate erythropoiesis, and improve recovery^4,12^.

Inherent to its sex steroid hormone role, testosterone supplementation has shown a wide range of effects on lipid metabolism with contrasted results. A few studies have reported a decrease in low-density lipoprotein (LDL) and total cholesterol levels following supplementation in postmenopausal women and hypogonadal men^13,14^. In contrast, others observed a significant reduction of circulating high-density lipoprotein (HDL) in female-to-male (FM) transsexuals under testosterone therapy, suggesting that testosterone might have an atherogenic effect over longer periods^15–17^.

Most of these studies investigated the effect of testosterone supplementation at the level of lipoproteins, total cholesterol, or total triglyceride content, which are not representative of the circulatory lipid diversity and do not allow for a deeper understanding of the testosterone role in the regulation of lipid metabolism. Each lipid class (e.g., glycerolipids, cholesterol esters, glycerophospholipids, sphingolipids) comprises a wide variety of chemically diverse species with varying fatty acid composition. Structurally closely related species can have different biological roles and be associated with distinct metabolic consequences^18^. Recent advances in mass spectrometry technology have allowed for detailed, omics-scale lipid analysis yielding multiparametric personalized profiles that reflect metabolic individuality, including the response to challenges, such as dietary regimen, drug treatment, or the level of physical activity. The analytical approach to exhaustively elucidate the lipid structural diversity and abundance in complex biological matrices, such as blood plasma, is designated as *lipidomics* and has emerged as next-generation profiling for metabolic health monitoring and to gain in-depth insights into lipid metabolism^19,20^. Measuring individual lipid species with high selectivity and fatty acid chain resolved composition will better understand lipids’ roles in maintaining health and the etiology of multiple pathophysiological processes.

Lipids are essential structural components of cells and play a pivotal role in various cellular processes. Apart from their vital function in energy production and storage, they also impact the functioning and occurrence of membrane proteins and signaling and inflammatory pathways^21–24^. Therefore, it is necessary to investigate changes in the lipidome and its regulation to gain a comprehensive understanding of physiological processes at the molecular level.

In the present study, the impact of low-dose testosterone gel administration on the plasma lipidome was investigated short-term, over a 28-day period, on matched healthy young female subjects (n = 14, premenopausal, aged 22–37) using a comprehensive and quantitative mass spectrometry (MS)-based lipid profiling.

## Material and methods

### Testosterone gel administration study

Fourteen healthy female subjects volunteered to participate in an open-label trial. A full description of patient inclusion and procedures is described elsewhere^25^. In short, the study was divided into three distinct 28-day phases corresponding to three consecutive menstrual cycles. The first phase corresponded to the control phase before testosterone treatment. During the second phase, volunteers self-administered 0.5 g of testosterone gel (Tostran® 20 mg/g) every morning on the upper thigh and/or abdomen (28 days). On visiting days, venous blood samples were obtained before administering the treatment, ensuring that each sample was collected around 24 h after the last application of the gel. The third phase corresponded to the 28-day post-treatment period. The study started on Monday following the onset of the menses, and the administration phase (phase 2) also started the week following their onset. Compliance was monitored by weighing the testosterone gel tube at each study visit. In case of menses delay, subjects had to wait one week until menstruation before starting the treatment. These requirements could generate slight heterogeneity between the subjects concerning the duration of their menstrual cycle. Over-the-counter and prescribed medication was recorded at each study visit. Serum samples were collected in 8.5-mL BD Vacutainer® SST™ II Advance tubes, and whole blood in 4-mL BD Vacutainer® K2EDTA Tubes. Serum and plasma were isolated and aliquoted following centrifugation at 1500 rpm for 15 min at 4 °C and stored at − 80 °C until analysis. All subjects provided written informed consent before any study procedures. The open-label trial was approved by the local Ethical Committee of the Canton de Vaud in Switzerland (2018-02106, SNCTP000003264) and Swissmedic (2018DR1168), registered on www.isrctn.com (ISRCTN10122130) and conducted in accordance with the Declaration of Helsinki.

### Serum hormones analysis

A panel of fourteen endogenous steroids was quantified in a 200-µL serum aliquot using a validated UPLC-MS/MS method^26^. Serum samples were spiked with 20 µL of an internal standard (IS) mix, and steroid hormones were extracted using supported liquid extraction on ISOLUTE SLE + (Biotage, Uppsala, Sweden) 400-µL 96-well plates. Following drying and reconstitution with mobile phase-suitable solvent, 10 µL of each extract was analyzed by ultra-high performance liquid chromatography (UHPLC) coupled to a Xevo-TQ-S triple quadrupole MS/MS system (Waters, Milford, MA, USA) operating in positive ionization mode. Chromatographic separation was carried out using an Ethylene Bridged Hybrid (BEH) C18 column (100 × 2.1 mm, 1.7 μm; Waters) equipped with a pre-column and set at 30 °C. Mobile phases A and B consisted of 0.1% formic acid in water and 0.1% formic acid in acetonitrile, respectively. The detailed instrumental conditions (chromatographic gradient, MRM transitions, ESI conditions, cone voltages, and collision energies) are described in Ponzetto et al.^26^. Extracts were analyzed again in negative ionization mode to quantify estrogens (estradiol, estrone, and estriol). Quantification was performed using an 8-point linear calibration model (weighting 1/x) in spiked depleted serum for each analyte. The limit of quantification (LOQ) for T was 20 pg/mL, DHT 50 pg/mL, progesterone 15 pg/mL, estradiol and estrone 5 pg/mL.

Serum luteinizing hormone (LH), follicular stimulating hormone (FSH), and sex-hormone binding globulin (SHBG) were measured by direct chemiluminescence using a Siemens ADVIA® Centaur™ Immunoassay System. The follicular phase was defined for estradiol < 81 pg/mL and progesterone < 1.6 ng/mL, combined with low FSH and LH. The ovulatory phase was characterized by LH peak (and/or LH higher than FSH) combined with estradiol ≥ 81 pg/mL and progesterone < 1.6 ng/mL. Finally, the luteal phase was defined as progesterone > 5.3 ng/mL. The days of the menses were also used to determine the menstrual phases accurately^27^.

Free testosterone was calculated using the Vermeulen method, with a standard average albumin concentration of 4.3 g/dL^28^.

### Deep targeted lipidomics

#### Lipid extraction

Plasma samples were thawed on ice, and lipids were extracted using a single-step extraction protocol (isopropanol-IPA)^29^. Briefly, 100 μL of IS mixture (containing 75 stable isotope-labeled species, the detailed composition is described in^30^) was added to 2 mL 96-deep-well plates and evaporated to dryness in an evaporator system (Turbovap 96, Biotage, Charlotte, NC, United States). The mixture consisted of UltimateSplash™ One, including 69 internal standards plus the following individual standards: L-Carnitine-(N-methyl-d3), Acetyl-L-carnitine-(N-methyl-d3), Butyryl-L-carnitine-(N-methyl-d3), O-Succinyl-L-carnitine-(N-methyl-d3), 3-Hydroxyisovaleryl-L-carnitine-(N-methyl-d3), Suberoyl-L-carnitine-(N-methyl-d3), Palmitoyl-L-carnitine-(N-methyl-d3), Stearoyl-L-carnitine-(N-methyl-d3), Oleoyl-L-carnitine-d3, Glucosyl(β) Ceramide(d18:1/15:0)-d7, Lactosyl(β) Ceramide(d18:1/15:0)-d7, dihydroceramide(d18:1/13:0)-d7, linoleic acid (18:2)-d4, arachidonic acid (20:4)-d8, docosahexanoic acid (22:6)-d5 and eicosapentaenoic acid (20:5)-d5 as described in^30^. The dried mixture was reconstituted with the addition of 25 μL of plasma samples and 125 μL of IPA, followed by incubation under agitation for 10 min at 1000 rpm and centrifugation at 20,000 g for 15 min at 4 °C. Finally, 75 μL of the supernatant was transferred to a new 96-well plate for UHPLC-MS/MS analysis. The addition of extraction solvent, incubation, and supernatant transfer were performed using a Bravo automated liquid-handling platform (Agilent Technologies, Santa Clara, California, USA).

#### UHPLC-MS/MS analyses

The plasma lipid extracts were analyzed by dual-channel UHPLC (Vanquish™ Duo, Thermo Scientific) coupled to a TSQ Altis triple-stage quadrupole mass spectrometer (Thermo Scientific) with electrospray ionization source operating in both negative and positive modes. As previously described, lipid analysis was carried out using an Acquity Premier BEH Amide column (1.7 µm, 100 mm × 2.1 mm I.D., Waters, Milford, MA, USA)^30^. Mobile phase A consisted of 10 mM ammonium acetate in acetonitrile:H_2_O (95:5; pH = 8.2), and mobile phase B was composed of 10 mM ammonium acetate in acetonitrile:H_2_O (50:50; pH = 7.4). The flow rate was 600 µL/min, column temperature 45 °C, sample injection volume 2 µL, and total analysis time 12 min (combining both ionization modes). The chromatography gradient, HESI source parameters, and optimized compound-dependent timed-selected reaction monitoring (t-SRM) conditions were previously described in detail^30^.

Lipid data were acquired in two steps consisting of a first initial screen of an extensive lipid panel in pooled plasma samples representative of the study population (one pool with baseline samples-D0 and one pool with samples at the peak of testosterone level-D45), followed by high-throughput quantification of filtered, robustly detectable lipid species. This primary screening was slightly modified from the original method^30^ comprising 1922 theoretical targets with the addition of 42 acylcarnitine species and 136 phospholipids (Tables S1 and S2) for a total of 2100 lipids targets (859 lipid species targeted in positive and 1241 in negative ionization). Following this initial screen, lipids fulfilling filtering criteria (intensity threshold and CV < 30% across QCs) were merged into a final targeted list for high-throughput quantification of plasma study extracts in positive and negative ionization modes. The subject-matched samples were analyzed in a randomized order together with pooled QC samples, injected every eight samples to correct for the MS-inherent signal intensity drift.

#### Data processing

Raw data files were processed using Xcalibur 4.1 and Trace Finder Clinical Research 4.0 from Thermo Fisher Scientific. The conditions across four consecutive injections of pooled samples to fulfill the filtering criteria were the following: relative standard deviation (RSD) of retention time (RSD < 5%), peak area (min. ion counts > 1000, and RSD < 30%), height (min. ion counts > 1000 and RSD < 30%) and lipid presence in at least 80% of replicates. Peak area integration was manually curated. Lipid concentrations were estimated as the peak area ratio between the analyte-selected reaction monitoring (SRM) and the most structurally similar IS SRM multiplied by its known spiked concentration. Signal intensity drift correction was performed using the LOWESS/Spline algorithm (span = 0.9), and lipid species with CV > 20% across QC pooled samples were removed from further statistical analysis. The correction for isotopic overlap based on lipid class separation by SRM was performed using the Shiny app of LICAR (https://slinghub.shinyapps.io/LICAR/)^31^.

### Statistical analysis

#### Steroid variation across time points

Non-parametric Kruskal–Wallis’ test followed by Dunn’s multiple comparison tests from the R package ‘PMCMRplus’ (‘kwAllPairsDunnTest’ function) was used to test differences between matching time points. Obtained *p* values were adjusted using the Bonferroni method.

#### Lipid profile clustering with t-distributed stochastic neighbor embedding (t-SNE) and hierarchical clustering

tSNE scatterplots of acquired lipid profiles (for each subject and across all time points) were generated after log2-transformation and z-score scaling using R package ‘Rtsne’ with perplexity and theta parameters set at 5 and 0.5, respectively^32^. Hierarchical clustering of subjects was performed using the ‘hclust’ R function on the log2-transformed lipid concentrations.

#### Lipid intra- and inter-individual variability

Coefficients of variation (CV) were calculated for each lipid species on non-imputed, untransformed datasets to avoid potential bias. Intra-individual variability was calculated as the CV across time points for each subject. In contrast, inter-individual variability was calculated as the CV between subjects for each time point and across the whole study period.

#### Non-linear models to identify altered lipids in response to testosterone administration

The lipid data were log-transformed before statistical analysis. The generalized least squares model (GLS) from R package ‘nmle’^33^ was used to investigate the fluctuation of lipid species over the study period at both the molecular and subclass level. The use of GLS allowed for the correlation between repeated measurements over time, providing a precise and accurate model of lipidomic data. The Benjamini–Hochberg method corrected the obtained *p* values for the false discovery rate (adjusted *p* < 0.05). The ‘*predict*’ function was then used to compute the predictive mean of significantly altered lipid species over time.

#### Pathway and enrichment analyses

Lipid ontology was performed using the online LION/web Lipid Ontology Enrichment software^34^. Log-transformed concentrations of lipid species were uploaded for ranked analysis using a one-tailed T-test between baseline and other study time points.

#### Correlation network analysis

The correlations between significantly altered lipids and hormone levels (testosterone, DHT, SHBG, estrone, estradiol) were first visualized with a corrplot from the ‘corrplot’ R package and further tested using the Pearson coefficient test. Statistical significance was determined at *P* < 0.05.

## Results

### General cohort characteristics and steroid profile

**Fig. 1:**
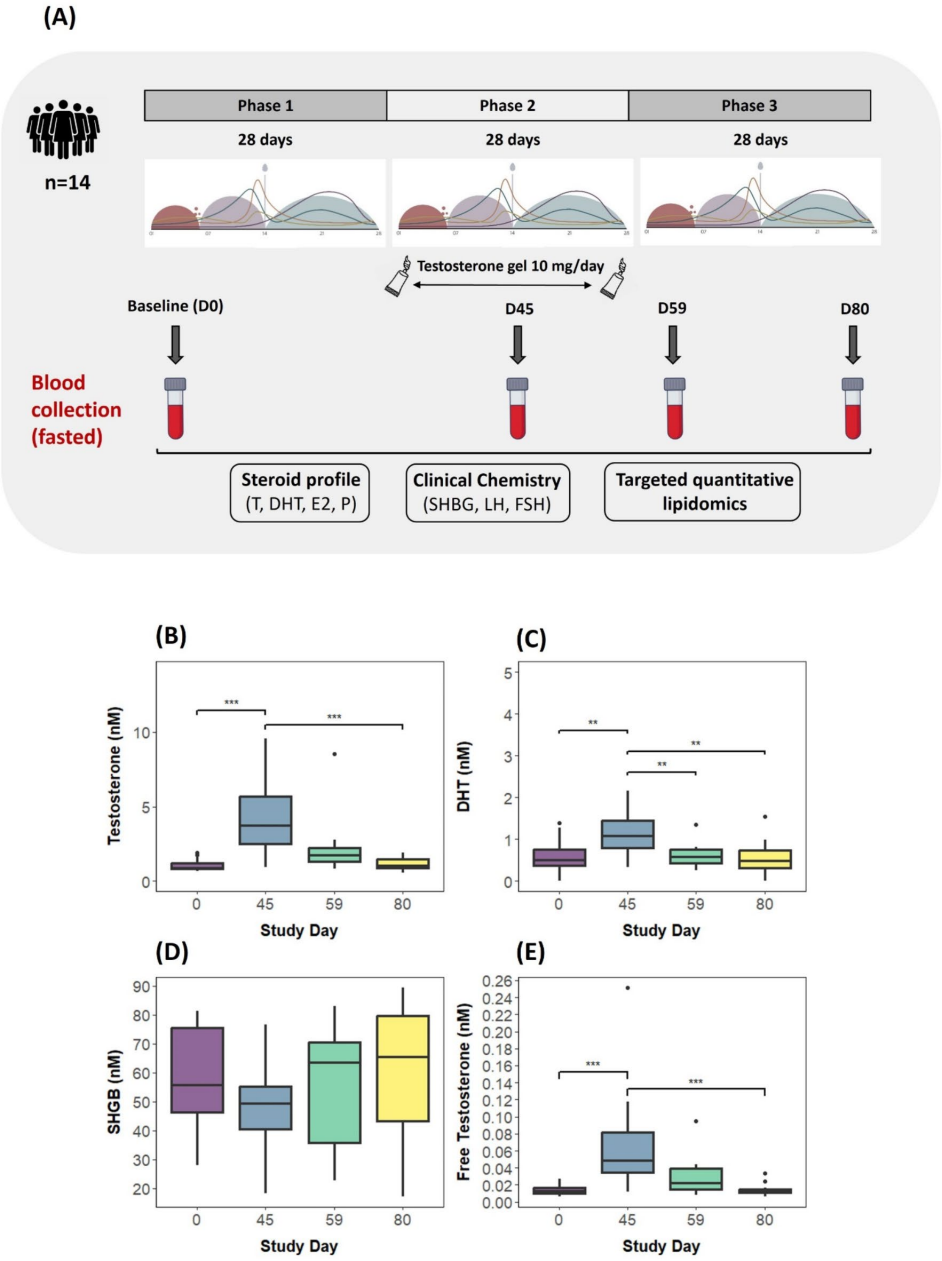
(**A**) Study design. Blood samples, including serum and plasma, were gathered at specific time points during the study: initially at baseline, then at 16 and 28 days following the start of daily testosterone treatment, and finally, at 23 days post-treatment completion. These samples underwent comprehensive analysis to assess steroid levels, clinical chemistry parameters, and the quantitative profiling of numerous lipid species, providing a multifaceted view of the subjects’ physiological responses. (**B**–**E**) Testosterone, dihydrotestosterone (DHT), SHBG, and free testosterone concentrations throughout the study (at selected time points (days). **p < 0.05 and ***p < 0.001.

The cohort was composed of 14 female individuals aged 22 to 37 years (mean = 28 ± SD 4.5), with a body mass index (BMI) of 21.4 ± 1.7 kg/m^2^ and regular menstruation cycle duration (29 ± 5 days). Table S3 provides detailed information about the subject’s baseline characteristics. Samples for quantitative lipidomic analysis were selected based on the fluctuation of testosterone levels throughout the study, as previously described^25^. The baseline sample corresponds to the first day of the study (D0) or baseline before treatment application, the second time point matches the testosterone peak during the administration phase (D45), the third time point corresponds to the end of the 28-day treatment phase (D59), and a final sample was taken at the end of the study or 24 days following the end of treatment (D80). Selected time points allowed for the assessment of the circulatory lipidome at different stages of the study, providing valuable insights into the effects of testosterone administration on lipid metabolism (Fig. 1A). Testosterone peak was observed on day D45 or 16 days after the testosterone gel application began. At this time point, the mean testosterone concentration (4.05 (± 2.41) nmol/L) was significantly higher compared to other time points with a fourfold change relative to baseline (Fig. 1B and Table S4). Free or bioavailable testosterone and dihydrotestosterone (DHT) as the main testosterone metabolite followed the same trend (Fig. 1C,E). Additionally, the SHBG levels showed the opposite trend, although the decrease at D45 compared to other time points was non-significant (Fig. 1D).

### Circulatory lipid diversity and abundance in apparently healthy young females

Among 2100 targeted lipid species (in the initial qualitative screen), 624 and 283 were detected in female plasma in positive and negative ionization mode, respectively. Following the quantification, the concentrations of a total of 597 lipids were robustly measured (with CV < 20% across QC samples) across the entire batch of samples (Fig. S1).

**Fig. 2:**
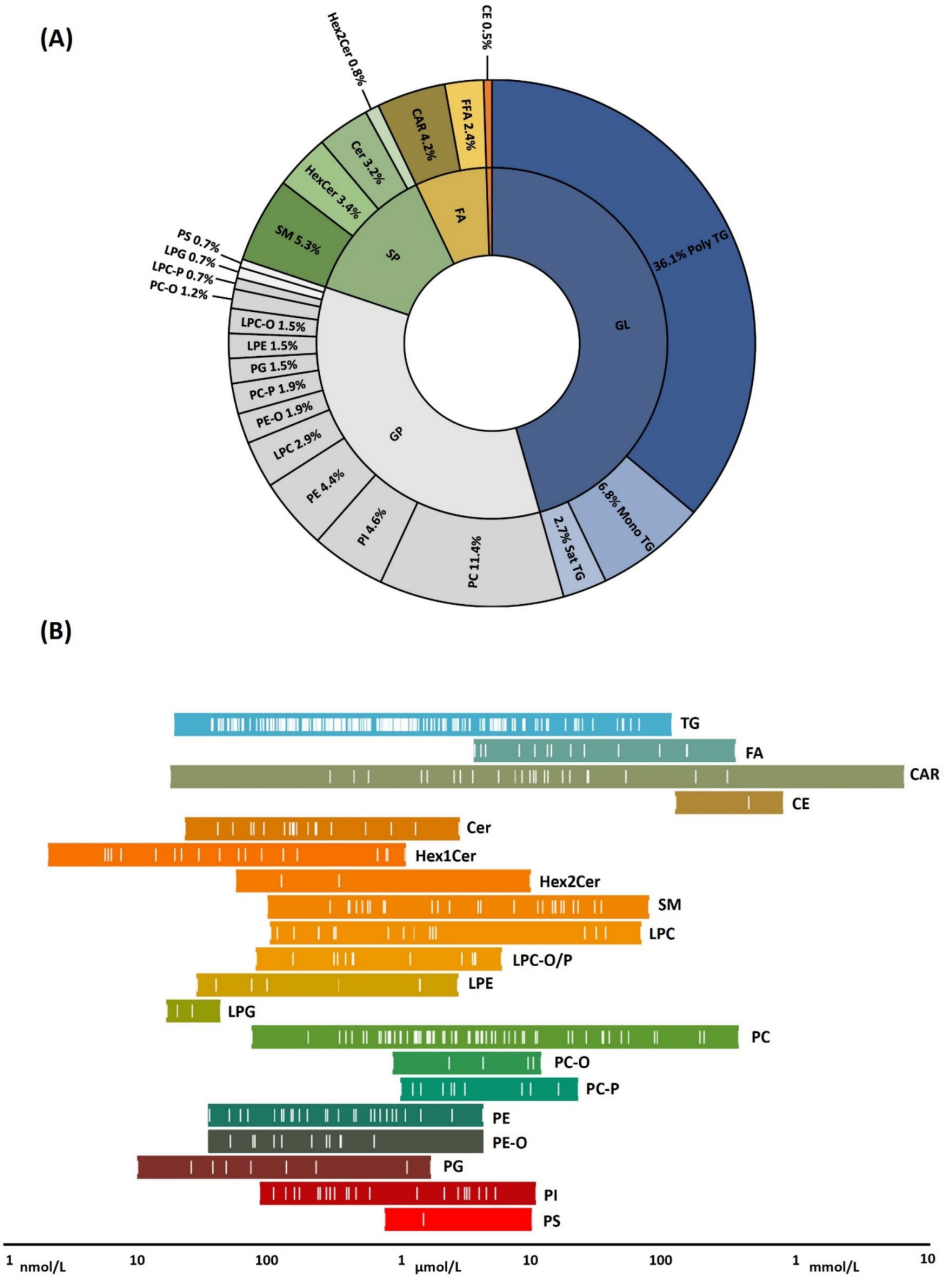
(**A**) Diversity and size of measurable plasma lipidome in healthy young females (**B**) Concentration range of measured lipid subclasses (based on baseline samples collected in the follicular phase). Lipid class abbreviations, TG, triglycerides; CAR, acylcarnitines; FA, free fatty acids; PC, phosphatidylcholine; PC-O, alkyl-phosphatidylcholines; PC-P, plasmanyl-phosphatidylcholines; LPC, lysophosphatidylcholine; LCP-O, alkyl-lysophosphatidylcholine; LPC-P, plasmanyl-lysophosphatidylcholine; PE, phosphatidyletanolamine; PE-O, alkyl-phosphatidyletanolamine; LPE, lysophosphatidyletanolamine; PG, phosphatidylglycerol; LPG, lysophosphatidylglycerol; PI, phosphatidylinositol; PS, phosphatidylserine; CL, cardiolipin; CER, ceramide; HexCer, hexosylceramide; Hex2Cer, trihexosylceramide; SM, sphingomyelin; CE, cholesteryl ester.

The structural diversity of the plasma lipidome was characterized using baseline samples of apparently healthy female subjects in the follicular phase of their menstrual cycle. A total of 22 lipid subclasses were reported (Fig. 2A) with the highest proportion of triacylglycerols (TG, 45.6%), followed by phosphatidylcholines (PC, 11.4%), sphingomyelins (SM, 5.3%), phosphatidylinositols (PI, 4.6%), phosphatidylethanolamines (PE, 4.4%), acylcarnitines (CAR, 4.2%) and other classes represented by less than 20 species. Polyunsaturated triacylglycerols (36.1%) were overrepresented compared to monounsaturated (6.8%) and saturated (2.7%) triacylglycerols. Measured concentrations of the analyzed plasma lipidome span seven orders of magnitude, from hexosylceramides (HexCer) present in nmol levels to highly abundant acylcarnitines (CAR) and cholesterol esters present in mmol levels (Fig. 2B). This large dynamic range was also observed within specific subclasses, such as CAR, TG, and PC, which span four to six orders of magnitude, depending on the species.

### Intra- and inter-individual variability of lipid signatures

**Fig. 3:**
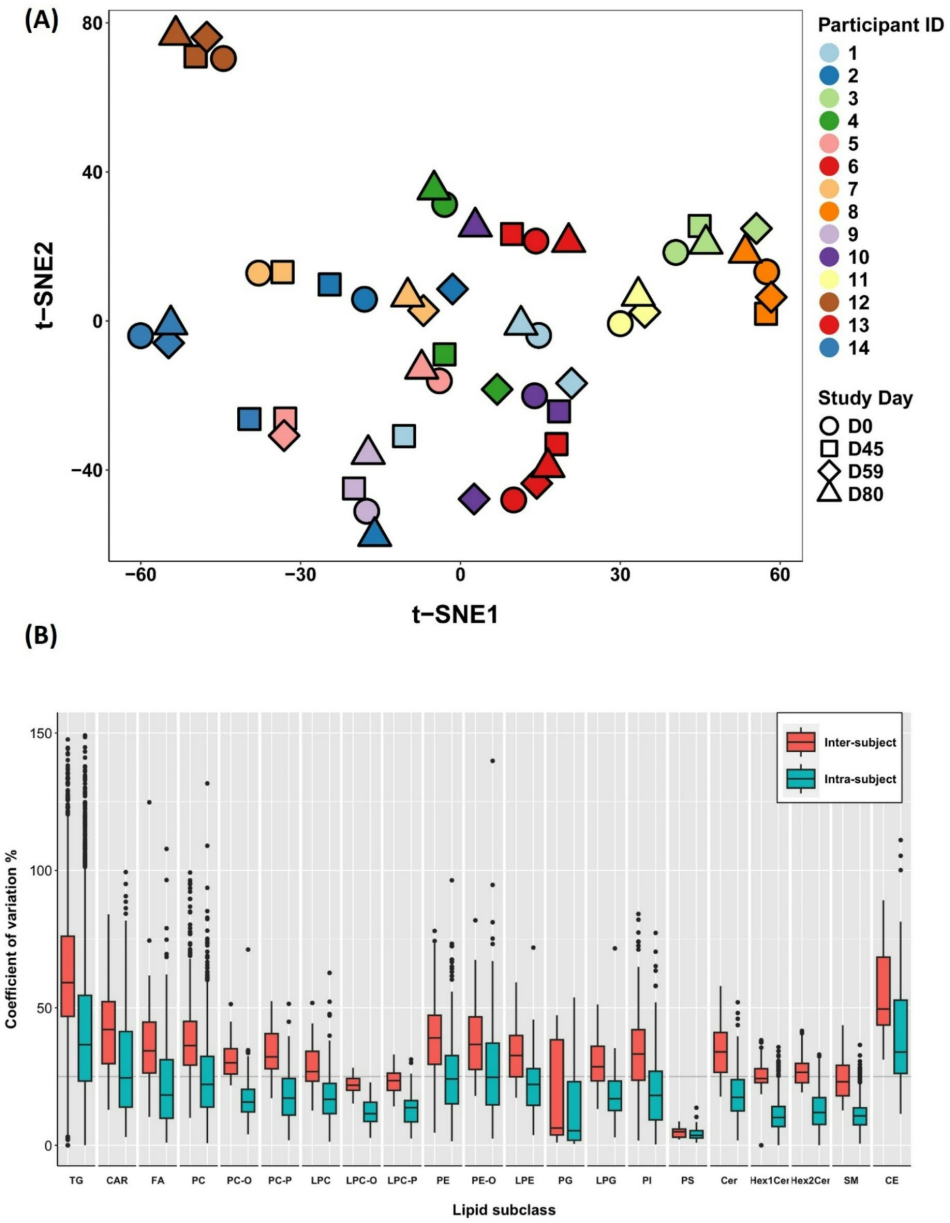
(**A**) t-SNE clustering of lipid signatures acquired from 14 female participants at different time points (**B**) Intra- and inter-individual variability of measured lipids within each class (considering all time points).

To further investigate the metabolic individuality of acquired lipid signatures or to which extent they are personalized or subject-specific (versus determined by the effect of treatment), we performed a t-SNE analysis. The t-SNE plot allowed us to visualize the clustering of multiparametric lipid signatures from all participants at all time points based on their similarity related to measured lipid concentration data (Fig. 3A). Hierarchical clustering analysis further confirmed these clustering results (Fig. S2). For more than half of the participants (8 out of 14), the lipid profiles acquired at different time points clustered together or nearby (regardless of the sampling time), while other individual signatures displayed higher heterogeneity and often clustered per two-time points (DO and D80 vs. D45 and D59). Most isolated or dispersed points (5 out of 14) correspond to the signatures recorded at the peak of testosterone concentration or D45. The obtained t-SNE clustering follows the computed intra- and inter-individual variability of lipid signatures per lipid (sub)class (Fig. 3B). The median intra-individual variability or variation over time within each subject was generally lower than inter-individual variability (considering all time points). The recorded intra-individual variability was below 25% for all lipid classes except for neutral lipids, TG, and CE, for which the estimated median intra- and inter-individual variability was above 30 and 50%, respectively. The inter-individual variation among different time points is comparable, apart from CE, which shows significant variation from one time point to another (Fig. S3).

### The effect of short-term testosterone treatment on circulatory lipid profile

**Fig. 4:**
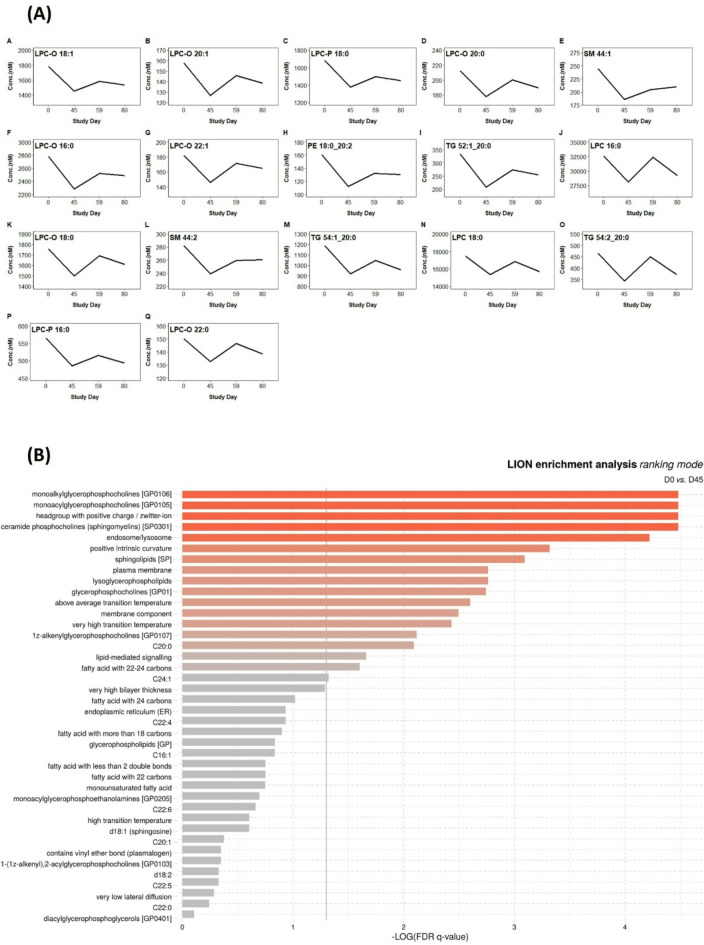
(**A**) Predictive mean estimated with the GLS model of the 17 significantly altered lipid species throughout the study and (**B**) lipid ontology enrichment analysis (LION) between baseline (D0) and the peak of testosterone level (D45).

The effect of testosterone administration was evaluated using the above-described time series assay (Fig. 1A) where D0 corresponds to baseline (before treatment), D45 to the testosterone peak during administration phase, D59 and D80 to three and 24 days, respectively, after the end of the treatment (Table S6). A significant decrease in the concentration of eleven different ether- and ester-linked saturated and monosaturated LPC species, two TG, two SM, and one PE was found at the peak of testosterone (D45, also coinciding with the significant decrease of SHBG) relative to baseline D0 (see Table S7 for effect size and *p* values computed with the nonlinear GLS model). The reduction for each lipid species ranged from 13 to 27% relative to baseline. This aligns with a general decreasing trend observed within LPC, LPC-O, and LPC-P classes at D45 relative to baseline (Table S5). No significant difference was observed for other time points. Using the GLS model, the predictive mean was plotted over time for the altered lipid species (Fig. 4A). To further elucidate alterations in lipid metabolism by testosterone administration, lipid ontology analysis was performed between baseline and D45 (Fig. 4B). According to enrichment analysis, the monoalkyl- and monaycl-LPC were the most significantly affected in D45 versus D0, as the GLS analysis output showed.

**Fig. 5:**
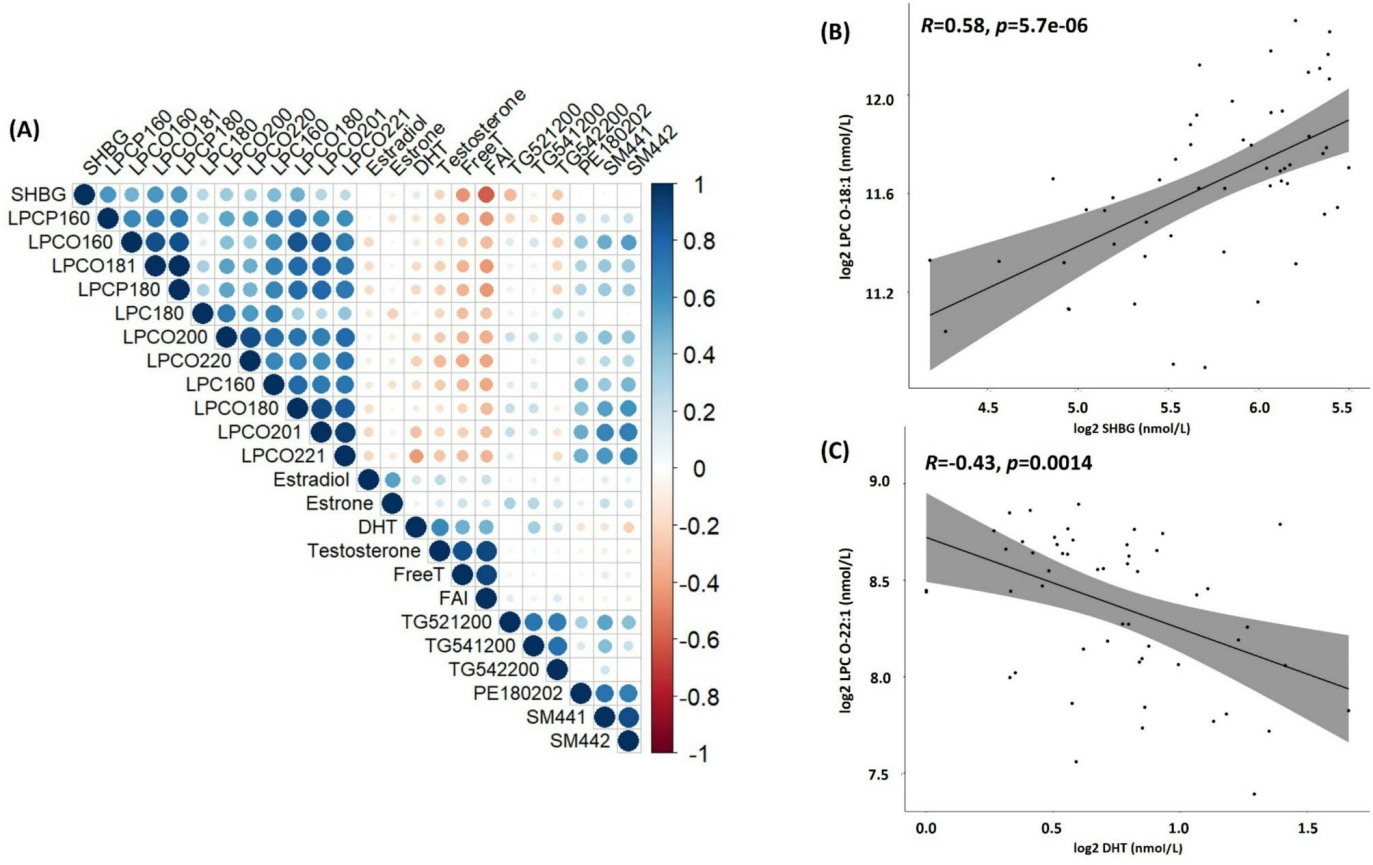
(**A**) Corrplot between the significantly altered plasma lipid species and the serum sex hormones concentrations. Non-blank cases indicate significant correlations, and the size of the circle is proportional to the coefficient of correlation while the color indicates either a positive (blue) or a negative (red) correlation. (**B**) Correlation between LPC-O-18:1 and SHBG concentrations and (**C**) between LPC-O-22:1 and DHT concentrations.

Interestingly, following this marked decrease at D45, during the post-treatment period (at D59 and D80), the lipid concentrations recovered but remained lower than the baseline levels (ns). To further investigate the relationship between lipids and steroids’ fluctuation, we have analyzed the correlation between the significantly affected lipid species and testosterone, DHT, SHBG, free T, free androgen index (FAI), estrone and estradiol (Fig. 5A). In line with the results of GLS modeling, strong positive correlations were observed between ester- and ether-linked LPC species and steroids.

Multiple ether-linked LPCs (LPC-O or alkyl-linked and LPC-P or alkenyl-linked or plasmalogens) and LPC 16:0 positively correlated with SHBG concentration, thus indirectly coinciding with free and total testosterone. For example, LPC O-18:1 exhibited the strongest positive correlation (r = 0.58; *p* < 0.001) with SHBG concentration (Fig. 5B) and simultaneously a significant negative correlation with free testosterone levels (r = − 0.34; *p* < 0.05). Specific noteworthy negative correlations were also observed between certain ether-linked LPCs such as LPC O-22:1 and DHT (Fig. 5C), the primary metabolite of testosterone and a potent agonist for the androgen receptor.

Importantly, the same lipid species also demonstrated significant negative correlations with the menstrual sex hormones estradiol and estrone and, to a lesser extent, progesterone. This finding suggests that, in addition to the effect of testosterone gel, fluctuations in sex hormones may also have an important effect on these lipid species.

## Discussion

The current study investigated the impact of testosterone gel administration on the plasma lipidome of healthy women. This is the first-of-its-kind longitudinal study that examines testosterone gel’s effects on women’s lipidome at the molecular level (over three menstrual cycles). The study aimed to shed light on the influence of androgen concentration on lipid metabolism in women, which remains poorly understood. Before investigating the effect of testosterone gel administration, we evaluated the composition and the intra- and inter-individual variability of measured circulatory lipidome in a cohort of apparently healthy young women (premenopausal, aged 22–37) not taking hormonal contraceptives. It has been recently recognized that structurally closely related lipid species can have different biological roles and be associated with distinct metabolic consequences^18^. Therefore, using the state-of-the-art deep-targeted methodology, we characterized the plasma lipidome with the highest specificity possible^30^. This approach confirmed the lipid-rich content (spanning from 2.03 in nM to 6.67 in mM) of human plasma represented by more than 20 lipid (sub)classes and, within each class, a panel of highly chemically diverse species with varying fatty acid chain composition^35–39^. The higher abundance and diversity of TAG and CE in plasma likely reflects liver lipid metabolism and lipid transport through the body^40^.

Collecting longitudinal samples over 80 days also allowed for evaluating biological variability within and between individuals. The intra-individual variability was strikingly low for most lipid subclasses, with a median lower than 25%, highlighting the tight regulation of the lipidome over time. The intra-subject variance was consistently and significantly lower than the inter-subject variance, implying that the circulatory lipid signatures are highly individualized or subject-specific despite the testosterone treatment.

These results indicate that individual-specific factors determine and have a greater impact on the lipidome composition than testosterone exposure. This assumption is further supported by Snyder et al., who demonstrated that lipid signatures are highly personalized, particularly when assessed longitudinally^32,41^. Additionally, the lack of clustering between time points suggested that the changes in the lipid profiles of healthy women during a 28-day testosterone gel administration study were minimal.

The effect of the administration of testosterone was evaluated over 28 days of treatment. During the administration phase, the abundance of most lipid subclasses decreased, suggesting lipid utilization or catabolism. Importantly, the testosterone peak coincided with a significant decrease in the levels of 17 lipid species with the highest effect on ether- and ester-linked lysophophatidylcholines (LPC). These lipid species exhibited a robust correlation, suggesting they are similarly impacted. Furthermore, these lipids demonstrated significant negative correlations with free and total testosterone and its primary metabolite, DHT, which is also a potent androgen receptor agonist. Additionally, a positive association with SHBG concentration, which also decreased as expected after androgen administration, was revealed, suggesting a potential mechanism linked to the bioavailability of testosterone^42^.

This subtle increase in testosterone levels is physiologically relevant, specifically if we draw a parallel to testosterone levels in women diagnosed with polycystic ovary syndrome (PCOS). The median testosterone concentration reported during the treatment period was above the upper limit of the healthy female range and was close to concentrations reported in females with PCOS^43–45^. PCOS is a common endocrine disorder that affects women of reproductive age and is associated with metabolic disturbances, including dyslipidemia. Investigating the impact of testosterone gel administration on the lipidome of healthy women with a comparable increase in testosterone levels to those observed in PCOS patients may provide insights into the potential role of testosterone in the development of dyslipidemia in PCOS. Multiple studies reported decreased levels of LPCs in females with confirmed PCOS compared to controls^46–49^. Due to the multifactorial nature of this syndrome, it is challenging to discriminate which features of PCOS contribute the most to this effect. Li et al. notably suggested that obesity in PCOS (typically accompanied by hyperinsulinemia) stimulates the production of bioactive lipids, while androgen has the opposite effect^49^. Elaborating further, LPC 16:0 was the only LPC with lower values in both lean and obese PCOS vs controls, suggesting that this downregulation could be related to hyperandrogenism with androgen overriding the stimulatory effects of insulin. Thus, our study further corroborates the hypothesis that an increase in androgen directly impacts LPC metabolism by decreasing their production.

These observations do not allow for extrapolation of the mechanism behind this phenomenon. Nevertheless, it is possible to generate several hypotheses regarding the influence of testosterone and androgen hormones on lipid metabolism, particularly LPC subclasses. An action of androgen on phospholipase (PL) activity or level may be hypothesized. Indeed, a study showed an inverse relationship between testosterone and PLA2 levels in testosterone-deficient patients^50^. In the current study, no significant changes in the PC/LPC ratio were observed at the testosterone peak, suggesting that the observed effect is not directly linked to PL activity. A link between PexRAP-Ether lipids-PPARγ and testosterone may also be postulated. Indeed, it was shown that testosterone inhibits the activity of PPARγ, which controls adipogenic differentiation and lipid metabolism^51^. In parallel, PexRAP, a protein required for alkyl ether lipid synthesis, is associated with PPARγ transcriptional activity^52^. Hence, a direct action of testosterone on PexRAP by repressing its activity may explain the decrease of alkyl ether LPC, although the alkyl ether PC was not significantly decreased. Further investigation of the underlying mechanism and potential physiological implications of described observations should be performed in model organisms.

While the study provides valuable insights into the effect of testosterone administration on the lipidome of healthy women, it also has some limitations that need to be considered. The main limitation is the relatively small sample size, which could potentially impact the statistical power of the analysis; however, the repeated measurements significantly enhance the statistical power, making the findings more robust and reliable.

The baseline timepoint was selected as the first plasma sample collected in the study, independently of menstrual cycle phase, with the objective of comparing lipid profiles under low endogenous steroid levels to those observed at peak testosterone concentration on day 45. While fluctuations in sex hormones across the menstrual cycle could contribute to lipid variability, our primary goal was to assess the impact of exogenous testosterone administration rather than intra-cycle changes. Furthermore, we cannot disentangle the potential effect of confounding factors, such as fluctuations of other sex hormones during the menstrual cycle. It is worth noting that many samples at D45 were collected during the luteal phase, while baseline samples were predominantly from the follicular phase. Although this could introduce variability in hormone levels, the cyclic fluctuations of estradiol remained stable despite testosterone administration as reported previously, reducing the likelihood of significant menstrual cycle effects on the lipid changes observed.

Aromatase activity plays a key role in converting androgens to estrogens, raising the possibility that testosterone administration could influence estradiol and estrone levels. However, in our study, we observed that estrone and estradiol continued to fluctuate in a cyclic manner despite testosterone administration, suggesting no significant increase in aromatase activity. This observation was already published previously^25^ and indicates that the associations observed are likely driven by the moderate increase in testosterone concentrations following transdermal administration, in addition to endogenous hormonal fluctuations, rather than increased conversion of testosterone to estrogens.

Finally, the effect of testosterone administration on lipid metabolism should be further investigated across different tissues, such as liver or adipose tissue, which could provide additional insights into the mechanism of action. Insulin sensitivity was not directly assessed in this study, but it is important to acknowledge that testosterone has been shown to influence insulin resistance, particularly in the context of its effects on adipose tissue. Although no significant increase of visceral adipose tissue was observed (data not shown), testosterone administration, especially via gel, may affect adipose tissue distribution and function, potentially leading to increased adipose tissue insulin resistance. This, in turn, could influence serum lipid composition, as insulin resistance is known to affect lipid metabolism. Future studies exploring the interaction between testosterone, insulin sensitivity, and lipid profiles would provide a more comprehensive understanding of the mechanisms underlying the changes in lipid species observed in this study.

## Conclusion

In summary, this study allowed for the characterization of the plasma lipidome of healthy female subjects not taking hormonal contraception throughout three menstrual cycles, in addition to the evaluation of the effect of testosterone gel administration on lipid metabolism. Using a robust and comprehensive quantitative method, the large diversity of lipid species was measured, and plasma lipid signatures showed high individuality. Further on, we revealed that administering testosterone gel alters plasma lipidome, particularly ether- and ester-linked LPC. These lipid species were significantly depleted at the peak of testosterone. Beyond LPC, the impact on plasma lipidome was negligible, thus emphasizing the presence of robust regulatory mechanisms that maintain lipid homeostasis in the body and the importance of understanding these mechanisms for developing targeted therapeutic interventions for diseases associated with lipid dysregulation.

## Electronic supplementary material

Below is the link to the electronic supplementary material.

Supplementary Material 1

Supplementary Material 2

## Data Availability

All data are available as supplements to the present manuscript.

## References

[1] Schiffer, L., Arlt, W. & Storbeck, K.-H. Intracrine androgen biosynthesis, metabolism and action revisited. *Mol. Cell. Endocrinol.* **465**, 4–26 (2018). 28865807 10.1016/j.mce.2017.08.016 PMC6565845

[2] Zumoff, B., Strain, G. W., Miller, L. K. & Rosner, W. Twenty-four-hour mean plasma testosterone concentration declines with age in normal premenopausal women. *J. Clin. Endocrinol. Metab.* **80**, 1429–1430 (1995). 7714119 10.1210/jcem.80.4.7714119

[3] Moreau, K. L., Babcock, M. C. & Hildreth, K. L. Sex differences in vascular aging in response to testosterone. *Biol. Sex Differ.* **11**, 18 (2020). 32295637 10.1186/s13293-020-00294-8 PMC7161199

[4] Hartgens, F. & Kuipers, H. Effects of androgenic-anabolic steroids in athletes. *Sports Med.* **34**, 513–554 (2004). 15248788 10.2165/00007256-200434080-00003

[5] Vegunta, S., Kling, J. M. & Kapoor, E. Androgen therapy in women. *J. Women’s Health* **29**, 57–64 (2020). 10.1089/jwh.2018.7494 31687883

[6] Goldstat, R., Briganti, E., Tran, J., Wolfe, R. & Davis, S. R. Transdermal testosterone therapy improves well-being, mood, and sexual function in premenopausal women. *Menopause* **10**, 390–398 (2003). 14501599 10.1097/01.GME.0000060256.03945.20

[7] Tuiten, A. et al. Time course of effects of testosterone administration on sexual arousal in women. *Arch. Gen. Psychiatry* **57**, 149–153 (2000) (**discussion 155-156**). 10665617 10.1001/archpsyc.57.2.149

[8] van Peer, J. M., Enter, D., van Steenbergen, H., Spinhoven, P. & Roelofs, K. Exogenous testosterone affects early threat processing in socially anxious and healthy women. *Biol. Psychol.* **129**, 82–89 (2017). 28811112 10.1016/j.biopsycho.2017.08.003

[9] Davis, S. R. Androgen therapy in women, beyond libido. *Climacteric* **16**(Suppl 1), 18–24 (2013). 23647457 10.3109/13697137.2013.801736

[10] Christou, M. A. et al. Effects of anabolic androgenic steroids on the reproductive system of athletes and recreational users: A systematic review and meta-analysis. *Sports Med.* **47**, 1869–1883 (2017). 28258581 10.1007/s40279-017-0709-z

[11] Hartgens, F. et al. Androgenic-anabolic steroid-induced body changes in strength athletes. *Phys. Sportsmed.* **29**, 49–65 (2001). 20086552 10.3810/psm.2001.01.316

[12] Bachman, E. et al. Testosterone induces erythrocytosis via increased erythropoietin and suppressed hepcidin: Evidence for a new erythropoietin/hemoglobin set point. *J. Gerontol. A Biol. Sci. Med. Sci.* **69**, 725–735 (2014). 24158761 10.1093/gerona/glt154 PMC4022090

[13] Fernández-Carvajal, J. et al. Lipid profile modifications in post-menopausal women treated with testosterone gel. *Endocrinol. Nutr. (Engl. Ed.)* **59**, 44–49 (2012). 10.1016/j.endonu.2011.07.010 22115702

[14] Zgliczynski, S. et al. Effect of testosterone replacement therapy on lipids and lipoproteins in hypogonadal and elderly men. *Atherosclerosis* **121**, 35–43 (1996). 8678922 10.1016/0021-9150(95)05673-4

[15] Chandra, P., Basra, S. S., Chen, T. C. & Tangpricha, V. Alterations in lipids and adipocyte hormones in female-to-male transsexuals. *Int. J. Endocrinol.* **2010**, 945053 (2010). 20706676 10.1155/2010/945053 PMC2913672

[16] Goh, H. H., Loke, D. F. & Ratnam, S. S. The impact of long-term testosterone replacement therapy on lipid and lipoprotein profiles in women. *Maturitas* **21**, 65–70 (1995). 7731386 10.1016/0378-5122(94)00861-z

[17] Robinson, G. A. et al. Sex hormones drive changes in lipoprotein metabolism. *iScience* **24**, 103257 (2021). 34761181 10.1016/j.isci.2021.103257 PMC8567005

[18] Carrard, J. et al. Metabolic view on human healthspan: A lipidome-wide association study. *Metabolites* **11**, 287 (2021). 33946321 10.3390/metabo11050287 PMC8146132

[19] Meikle, T. G., Huynh, K., Giles, C. & Meikle, P. J. Clinical lipidomics: Realizing the potential of lipid profiling. *J. Lipid Res.* **62**, 100127 (2021). 34582882 10.1016/j.jlr.2021.100127 PMC8528718

[20] Tumanov, S. & Kamphorst, J. J. Recent advances in expanding the coverage of the lipidome. *Curr. Opin. Biotechnol.* **43**, 127–133 (2017). 27915214 10.1016/j.copbio.2016.11.008 PMC5312421

[21] Chiurchiù, V., Leuti, A. & Maccarrone, M. Bioactive lipids and chronic inflammation: Managing the fire within. *Front. Immunol.* **9**, 38 (2018). 29434586 10.3389/fimmu.2018.00038 PMC5797284

[22] Rose, T. D. et al. Lipid network and moiety analysis for revealing enzymatic dysregulation and mechanistic alterations from lipidomics data. *Brief Bioinform.* **24**, bbac572 (2023). 36592059 10.1093/bib/bbac572 PMC9851308

[23] Allen, J. A., Halverson-Tamboli, R. A. & Rasenick, M. M. Lipid raft microdomains and neurotransmitter signalling. *Nat. Rev. Neurosci.* **8**, 128–140 (2007). 17195035 10.1038/nrn2059

[24] Serhan, C. N., Chiang, N. & Van Dyke, T. E. Resolving inflammation: Dual anti-inflammatory and pro-resolution lipid mediators. *Nat. Rev. Immunol.* **8**, 349–361 (2008). 18437155 10.1038/nri2294 PMC2744593

[25] Salamin, O. et al. Longitudinal evaluation of multiple biomarkers for the detection of testosterone gel administration in women with normal menstrual cycle. *Drug Test. Anal.*10.1002/dta.3040 (2021). 33817997 10.1002/dta.3040

[26] Ponzetto, F. et al. Longitudinal monitoring of endogenous steroids in human serum by UHPLC-MS/MS as a tool to detect testosterone abuse in sports. *Anal. Bioanal. Chem.* **408**, 705–719 (2016). 26677027 10.1007/s00216-015-9185-1

[27] Schulze, J. et al. Urinary steroid profile in relation to the menstrual cycle. *Drug Test. Anal.* **13**, 550–557 (2021). 33142032 10.1002/dta.2960 PMC7984021

[28] Vermeulen, A., Verdonck, L. & Kaufman, J. M. A critical evaluation of simple methods for the estimation of free testosterone in serum. *J. Clin. Endocrinol. Metab.* **84**, 3666–3672 (1999). 10523012 10.1210/jcem.84.10.6079

[29] Medina, J. et al. Single-step extraction coupled with targeted HILIC-MS/MS approach for comprehensive analysis of human plasma lipidome and polar metabolome. *Metabolites* **10**, 495 (2020). 33276464 10.3390/metabo10120495 PMC7760228

[30] Medina, J. et al. Omic-scale high-throughput quantitative LC-MS/MS approach for circulatory lipid phenotyping in clinical research. *Anal. Chem.* **95**, 3168–3179 (2023). 36716250 10.1021/acs.analchem.2c02598

[31] Cazenave-Gassiot, A. et al. LICAR: An application for isotopic correction of targeted lipidomic data acquired with class-based chromatographic separations using multiple reaction monitoring. *Anal. Chem.* **93**, 3163–3171 (2021). 33535740 10.1021/acs.analchem.0c04565

[32] Contrepois, K. et al. Molecular choreography of acute exercise. *Cell* **181**, 1112-1130.e16 (2020). 32470399 10.1016/j.cell.2020.04.043 PMC7299174

[33] Harrell, F. E. Modeling longitudinal responses using generalized least squares. In *Regression Modeling Strategies: With Applications to Linear Models, Logistic and Ordinal Regression, and Survival Analysis* (ed. Harrell, F. E., Jr.) 143–160 (Springer, 2015). 10.1007/978-3-319-19425-7_7.

[34] Molenaar, M. R. et al. LION/web: A web-based ontology enrichment tool for lipidomic data analysis. *GigaScience* **8**, giz061 (2019). 31141612 10.1093/gigascience/giz061 PMC6541037

[35] Quehenberger, O. et al. Lipidomics reveals a remarkable diversity of lipids in human plasma1. *J. Lipid Res.* **51**, 3299–3305 (2010). 20671299 10.1194/jlr.M009449 PMC2952570

[36] Rampler, E. et al. A novel lipidomics workflow for improved human plasma identification and quantification using RPLC-MSn methods and isotope dilution strategies. *Anal. Chem.* **90**, 6494–6501 (2018). 29708737 10.1021/acs.analchem.7b05382

[37] Tabassum, R. et al. Genetic architecture of human plasma lipidome and its link to cardiovascular disease. *Nat. Commun.* **10**, 4329 (2019). 31551469 10.1038/s41467-019-11954-8 PMC6760179

[38] Sousa, B. C. et al. Comprehensive lipidome of human plasma using minimal sample manipulation by liquid chromatography coupled with mass spectrometry. *Rapid Commun. Mass Spectrom.* **9999**, e9472 (2023). 10.1002/rcm.9472 PMC12062770 36652341

[39] Ryan, M. J. et al. Comprehensive lipidomic workflow for multicohort population phenotyping using stable isotope dilution targeted liquid chromatography-mass spectrometry. *J. Proteome Res.* **22**, 1419–1433 (2023). 36828482 10.1021/acs.jproteome.2c00682 PMC10167688

[40] Loef, M. et al. Reproducibility of targeted lipidome analyses (Lipidyzer) in plasma and erythrocytes over a 6-week period. *Metabolites* **11**, 26 (2021). 10.3390/metabo11010026 PMC7823270 33396510

[41] Hornburg, D. et al. Dynamic lipidome alterations associated with human health, disease and ageing. *Nat. Metab.* **5**, 1578–1594 (2023). 37697054 10.1038/s42255-023-00880-1 PMC10513930

[42] Toscano, V. et al. Steroidal and non-steroidal factors in plasma sex hormone binding globulin regulation. *J. Steroid Biochem. Mol. Biol.* **43**, 431–437 (1992). 1390292 10.1016/0960-0760(92)90081-s

[43] Bui, H. N. et al. Testosterone, free testosterone, and free androgen index in women: Reference intervals, biological variation, and diagnostic value in polycystic ovary syndrome. *Clin. Chim. Acta* **450**, 227–232 (2015). 26327459 10.1016/j.cca.2015.08.019

[44] Clark, R. V. et al. Large divergence in testosterone concentrations between men and women: Frame of reference for elite athletes in sex-specific competition in sports, a narrative review. *Clin. Endocrinol.* **90**, 15–22 (2019). 10.1111/cen.13840 30136295

[45] Handelsman, D. J., Hirschberg, A. L. & Bermon, S. Circulating testosterone as the hormonal basis of sex differences in athletic performance. *Endocr. Rev.* **39**, 803–829 (2018). 30010735 10.1210/er.2018-00020 PMC6391653

[46] Haoula, Z. et al. Lipidomic analysis of plasma samples from women with polycystic ovary syndrome. *Metabolomics* **11**, 657–666 (2015). 25972770 10.1007/s11306-014-0726-y PMC4419155

[47] Sun, Z. et al. Identification of potential metabolic biomarkers of polycystic ovary syndrome in follicular fluid by SWATH mass spectrometry. *Reprod. Biol. Endocrinol.* **17**, 45 (2019). 31186025 10.1186/s12958-019-0490-y PMC6560878

[48] Zhang, Z. et al. Differential lipidomic characteristics of children born to women with polycystic ovary syndrome. *Front. Endocrinol.* **12**, 698734 (2021). 10.3389/fendo.2021.698734 PMC8380809 34434168

[49] Li, S. et al. Discovery of novel lipid profiles in PCOS: Do insulin and androgen oppositely regulate bioactive lipid production?. *J. Clin. Endocrinol. Metab.* **102**, 810–821 (2017). 27886515 10.1210/jc.2016-2692 PMC5477809

[50] Keleşoğlu, M. et al. The relationship between lipoprotein-associated phospholipase A2 with cardiovascular risk factors in testosterone deficiency. *Turk. J. Urol.* **44**, 103–108 (2018). 29511577 10.5152/tud.2017.30633 PMC5832369

[51] Cao, J., Maowulieti, G. & Yu, T. Effect of testosterone on the expression of PPARγ mRNA in PCOS patients. *Exp. Ther. Med.* **17**, 1761–1765 (2019). 30783446 10.3892/etm.2018.7101 PMC6364224

[52] Lodhi, I. J. et al. Inhibiting adipose tissue lipogenesis reprograms thermogenesis and PPARγ activation to decrease diet-induced obesity. *Cell Metab.* **16**, 189–201 (2012). 22863804 10.1016/j.cmet.2012.06.013 PMC3467338

