# Supplementary material for: Circulatory lipid signature in response to short-term testosterone gel treatment of healthy young females
Source: Sci Rep. 2025 Mar 29;15:10870. doi: 10.1038/s41598-025-92690-6 (PMC11955001; doi:10.1038/s41598-025-92690-6)
Supplement: Supplementary file 2 — Supplementary Material 2 [file 41598_2025_92690_MOESM2_ESM.pdf]

Supplementary data

Lipidomic Analysis of Healthy Women: Effects of Testosterone Gel Administration

Table of contents

Figure S1 – From initial screen to quantitative data and statistical analyses

Figure S2 – Hierarchical cluster of subjects according to their circulatory lipid levels

Figure S3 – Inter-subject coefficient of variation for each lipid subclass in function of the time points of the study.

Table S1 – SRM list for acylcarnitines added in the method

Table S2 – SRM list of additional phospholipids added in the method

Table S3 – Subject data demographics

Table S4 – Steroid concentrations

Table S5 - Subclass sum composition

Table S6 – GLS output

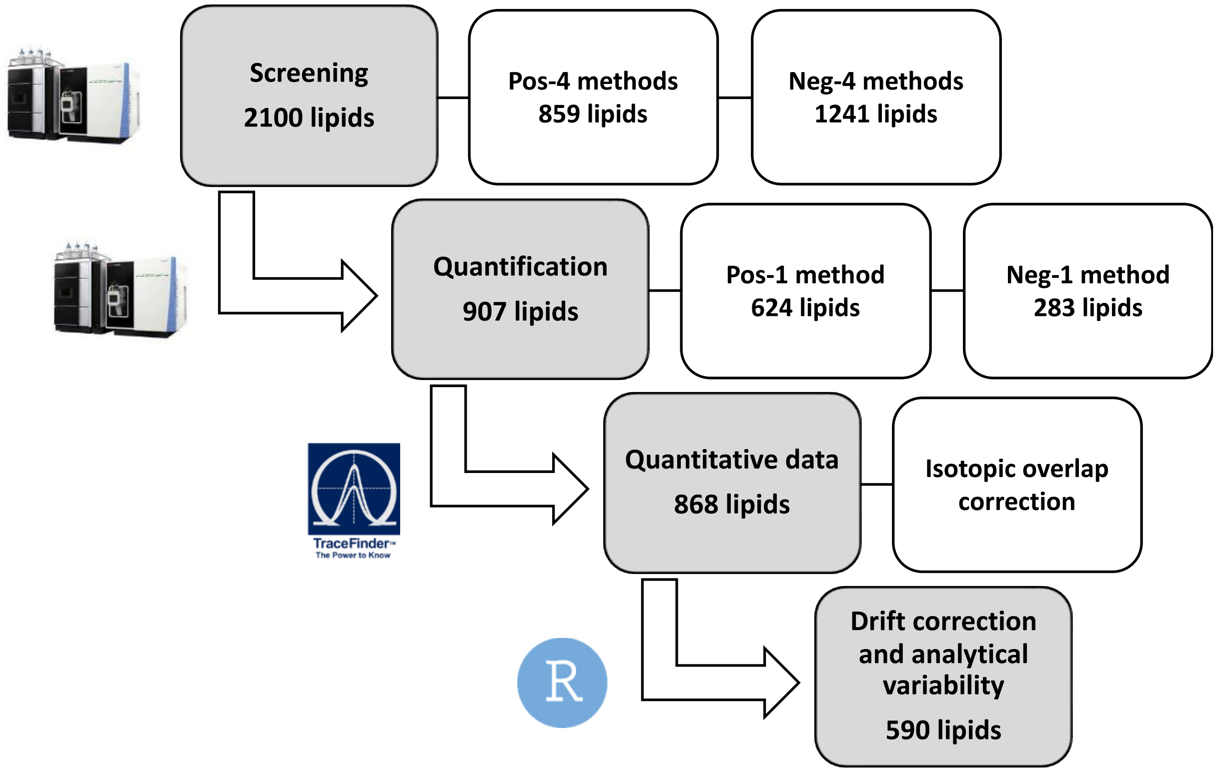

Figure S1. From initial screen to quantitative data and statistical analyses to investigate the effect of testosterone administration on the plasma lipidome.

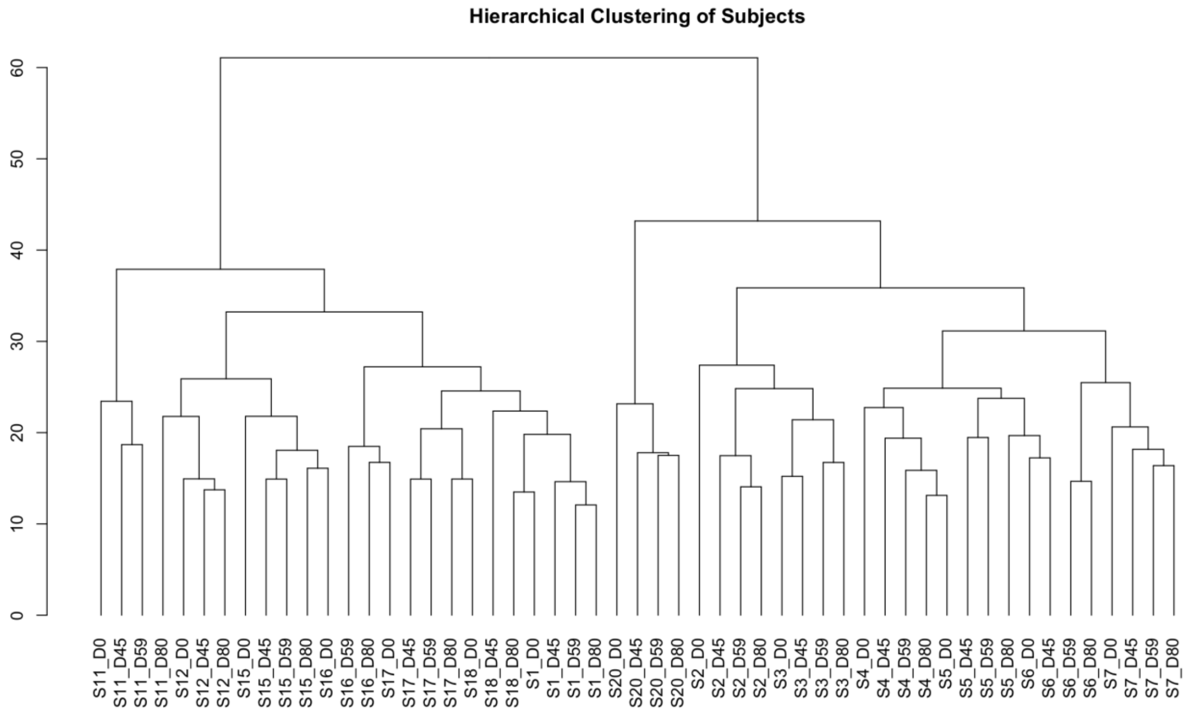

Figure S2. Hierarchical clustering of the subjects using lipid data.

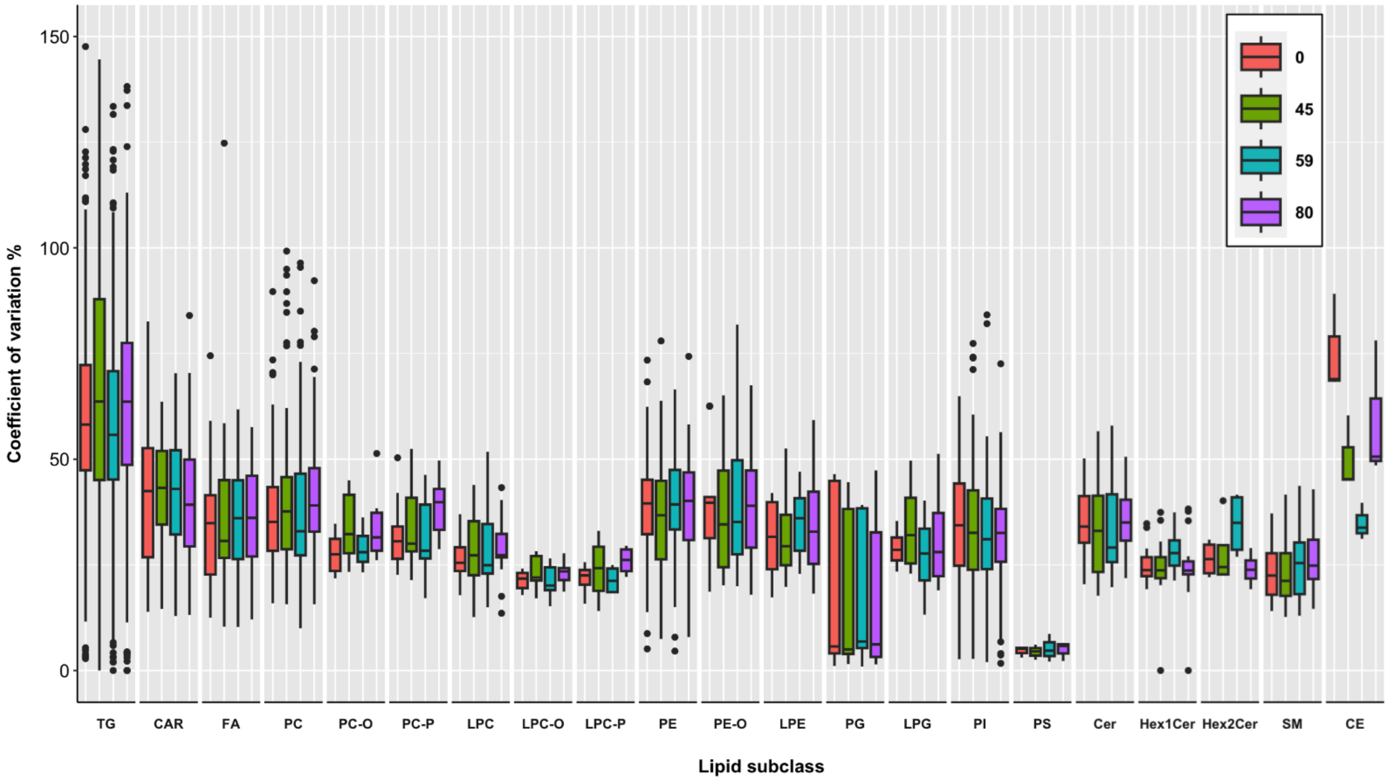

Figure S3. Inter-subject coefficient of variation for each lipid subclass in function of the time points of the study.
